# MiR-218 targets MeCP2 and inhibits heroin seeking behavior

**DOI:** 10.1038/srep40413

**Published:** 2017-01-11

**Authors:** Biao Yan, Zhaoyang Hu, Wenqing Yao, Qiumin Le, Bo Xu, Xing Liu, Lan Ma

**Affiliations:** 1 The State Key Laboratory of Medical Neurobiology, Institutes of Brain Science and The Collaborative Innovation Center for Brain Science, Fudan University, 138 Yixueyuan Road, Shanghai 200032, China

## Abstract

MicroRNAs (miRNAs) are a class of evolutionarily conserved, 18–25 nucleotide non-coding sequences that post-transcriptionally regulate gene expression. Recent studies implicated their roles in the regulation of neuronal functions, such as learning, cognition and memory formation. Here we report that miR-218 inhibits heroin-induced behavioral plasticity. First, network propagation-based method was used to predict candidate miRNAs that played potential key roles in regulating drug addiction-related genes. Microarray screening was also carried out to identify miRNAs responding to chronic heroin administration in the nucleus accumbens (NAc). Among the collapsed miRNAs, top-ranked miR-218 was decreased after chronic exposure to heroin. Lentiviral overexpression of miR-218 in NAc could inhibit heroin-induced reinforcement in both conditioned place preference (CPP) test and heroin self-administration experiments. Luciferase activity assay indicated that miR-218 could regulate 3′ untranslated regions (3′ UTR) of multiple neuroplasticity-related genes and directly target methyl CpG binding protein 2 (Mecp2). Consistently, *Mecp2*^*308/y*^ mice exhibited reduced heroin seeking behavior in CPP test. These data reveal a functional role of miR-218 and its target, MeCP2, in the regulation of heroin-induced behavioral plasticity.

Drug addiction is a psychiatric disorder characterized by loss of control over drug consumption and compulsive drug taking despite serious negative consequences^1^. Addictive drugs mediate their reinforcing properties by targeting the mesocorticolimbic dopaminergic (DA) circuitry, which contain the nucleus accumbens (NAc), the ventral tegmental area (VTA), prefrontal cortex (PFC) and hippocampus^2^. Drugs of abuse induce long-term adaptions in neuronal plasticity^3^, which is regulated by persistent alterations in gene expression. Extensive studies support that signaling cascades that regulate gene expression play fundamental roles in drug-induced neuroadaptions^4^,^5^,^6^,^7^. However, post-transcriptional regulation processes involved in drug addiction are largely unknown.

Recent studies indicate that epigenetic regulation of gene expression plays an important role in neurogenesis, synaptic plasticity and neurological disorders^8^,^9^,^10^. MicroRNAs (miRNAs) are a class of evolutionarily conserved, 18–25 nucleotide non-coding sequences that post-transcriptionally regulate gene expression. A miRNA may modulate the expression of hundreds of genes, either by translational suppression, or by degrading mRNAs that contains complementary sequences in the 3′ UTR^11^,^12^,^13^. Bioinformatic approaches indicate that miRNAs are likely to form miRNA-regulated gene networks, by preferentially targeting genes of certain pathways^14^. Recent studies indicate that several psychostimulants regulate miRNA expression in the NAc as well as other regions of mesocorticolimbic DA system, and manipulations of some specific miRNAs could alter the drug related behaviors and drug induced neuroplasticity^15^,^16^,^17^,^18^. But the role of miRNAs in heroin seeking behaviors, and the specific targets of key regulatory miRNAs remain unexplored.

In this study, we utilized bioinformatic approaches to predict potential key regulators in drug addiction. Among the top-ranked miRNAs, we found that miR-218 is down-regulated in response to chronic heroin administration. Lentiviral-mediated miR-218 overexpression significantly attenuated heroin-induced reinforcement in both conditioned place preference (CPP) and self-administration (SA) model. These effects were proposed to be mediated by suppression of target genes such as Mecp2, that participates in epigenetic control of gene transcription. Our observation provides a possible miRNA-mediated epigenetic regulatory mechanism in heroin addiction.

## Results

### Prediction of addiction-related miRNAs using network propagation-based strategy

**Figure 1:**
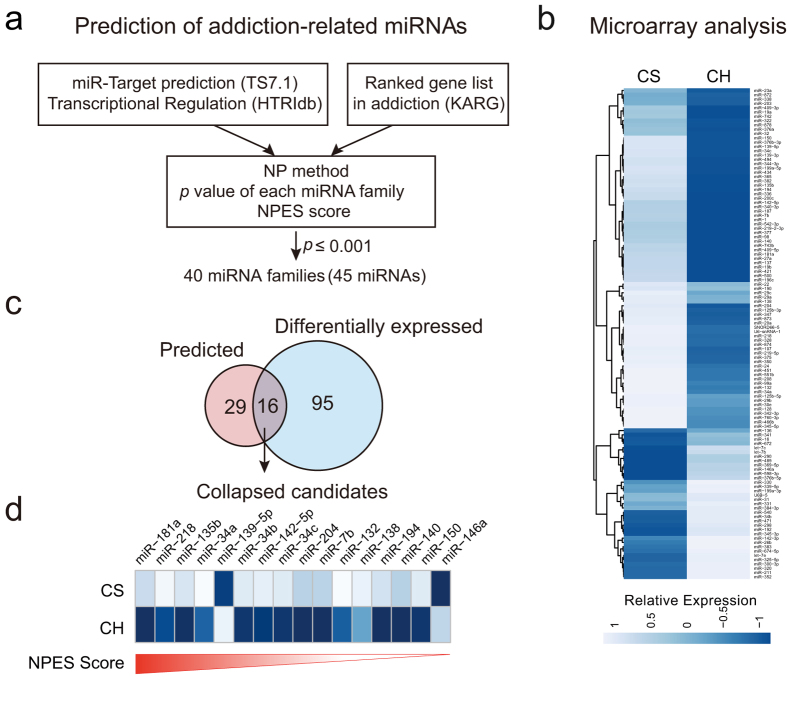
Screen for miRNAs regulated by chronic heroin administration. (**a**) Identification of addiction-related miRNA candidates. Network propagation based method (NP method) was used to predict potentially perturbed miRNAs in addiction-related process. Based on significance of NPES score, 40 miRNA families (45 miRNAs) were identified as putative candidates. (**b**) Heatmap of differentially expressed miRNAs in microarray. Rats were treated with saline or heroin (1 mg/kg, i.p., b.i.d.) for 7 days. MiRNAs that exhibit ≥25% alterations were shown. (**c**) NP method-based prediction and altered miRNAs in response to chronic heroin exposure was collapsed, resulting in 16 intersected miRNAs. (**d**) Heatmap of the 16 intersected miRNAs arranged by prediction scores (CS, Chronic Saline; CH, Chronic Heroin).

First, we tried to screen for miRNAs involved in drug addiction-related regulatory networks. A miRNA could regulate gene expression either by directly targeting drug addiction-related genes, or by targeting regulatory elements (e.g. transcription factors) whose impact may propagate across the whole regulatory network. Thus we utilized a network propagation-based model (NP-method)^19^ to predict miRNAs, whose target genes may contribute to major alterations in addiction-related pathways. We used miRNA-target regulation information (TargetScan 7.1) and the transcription regulatory database (HTRIdb) to model network effects of the miRNA perturbation, normalized reliability score from Knowledgebase for Addiction-Related Genes (KARG) to assess the potential involvement of addiction-related genes. The correlation between network effects of the miRNA perturbation and gene ranking was evaluated (Fig. 1a), which revealed 40 addiction-related miRNA families significantly enriched in regulation of addiction-related process (Table S1), among which, miR-132/212, miR-9, miR-181 *etc.* were top-ranked and previously reported^17^,^20^,^21^,^22^, indicating that NP-method is efficient in identifying miRNAs involved in addiction-related process.

### MiR-218 is down-regulated in response to chronic heroin administration

Microarray screening was carried out to identify miRNAs responding to chronic heroin administration. As shown in Fig. 1b, chronic treatment of heroin (CH, 1 mg/kg heroin, twice daily intraperitoneal injections for 7 days) resulted in differential expression (≥25%) of 111 miRNAs in the NAc, as compared with chronic saline group (CS, equivalent volume of saline, twice daily intraperitoneal injections for 7 days). The results obtained using NP-method and microarray screening were collapsed, resulting in 16 miRNAs from 14 families (Fig. 1d), which are likely important regulators of heroin-induced reinforcement.

**Figure 2:**
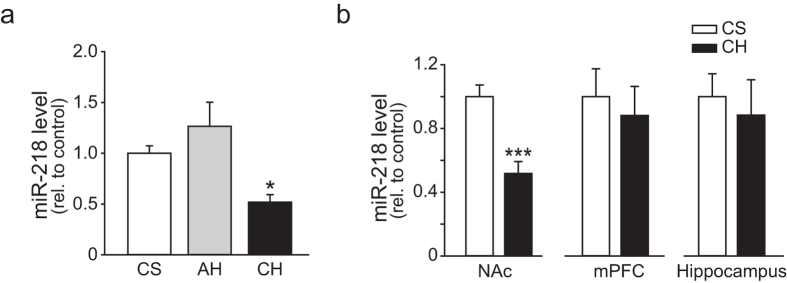
Chronic heroin administration downregulates miR-218 expression level in NAc. (**a**) Relative miR-218 level in NAc after saline, acute heroin or chronic heroin administration. miR-218 was significantly downregulated by chronic heroin exposure (CS, Chronic Saline, n = 10; AH, Acute Heroin, n = 6; CH, Chronic Heroin, n = 6; One-way ANOVA, *P < 0.05 *vs.* Saline group). (**b**) Relative miR-218 level in NAc, mPFC and hippocampus after saline or chronic heroin administration. MiR-218 level was decreased in NAc, but not in mPFC or hippocampus after chronic heroin administration (CS, Chronic Saline, n = 10; CH, Chronic Heroin, n = 6, Student’s *t*-test, ***P < 0.001). Data are expressed as mean ± s.e.m.

Among these 16 candidate miRNAs, miR-181a, a miRNA previously implicated in cocaine-induced plasticity^17^, was top-ranked. MiR-218, whose role in drug addiction has not been reported, was second highest scored, and microarray analysis indicated that miR-218 was down regulated in response to chronic heroin exposure (Fig. 1d). Quantitative real-time PCR also confirmed that chronic, but not acute heroin administration (AH, saline twice daily intraperitoneal injections for 7 days, then one injection of heroin, 1 mg/kg), significantly reduced the expression level of miR-218 in NAc (Fig. 2a). Moreover, the decrease of miR-218 level induced by chronic heroin exposure was restricted to NAc, since no significant change of miR-218 level was observed in mPFC or hippocampus (Fig. 2b).

### MiR-218 regulates heroin reinforcement

**Figure 3:**
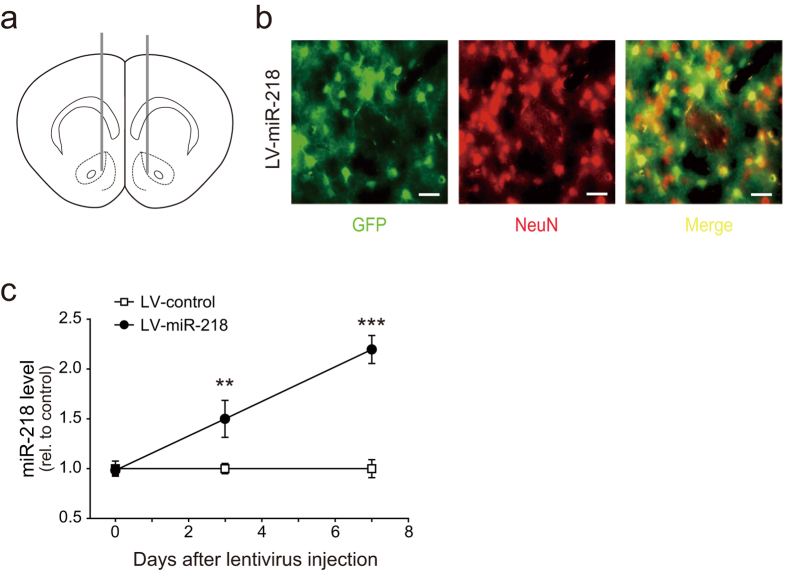
Lentiviral-mediated overexpression of miR-218 in NAc. (**a**) Schematic representation of lentivirus injection sites. (**b**) Cell localization of LV-miR-218 infected cells (green), neurons using NeuN antibody (red), scale bar = 20 μm. (**c**) Relative miR-218 level in NAc with LV-control or LV-miR-218 injection on day 0, 3, 7. Enhanced expression level of miR-218 after LV-miR-218 injection in NAc in day 3 and day 7 (LV-control, n = 5, LV-miR-218, n = 5 on each day, **P < 0.01; ***P < 0.001). Data are shown as mean ± s.e.m.

**Figure 4:**
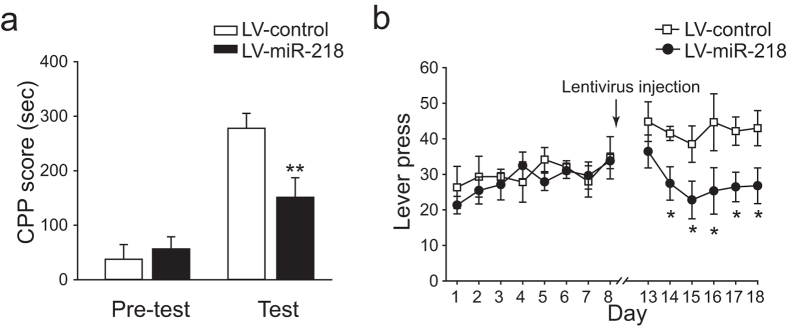
MiR-218 inhibits heroin seeking behavior. (**a**) CPP score of LV-control and LV-miR-218 group in pre-test and test session. Rats were subjected to LV-control or LV-miR-218 injection. Seven days later, they were subjected to pre-test, three conditioning sessions and CPP test. Heroin induced CPP is decreased in LV-miR-218 group (LV-control, n = 10, LV-miR-218, n = 9, **P < 0.01 *vs.* LV control during test session). (**b**) Lever presses of LV-control and LV-miR-218 rats in heroin self-administration. Rats were subjected to heroin self-administration procedure for eight days and then randomly grouped to receive NAc LV-control or LV-miR-218 injection. Three days later, daily SA sessions were resumed. LV-miR-218 group showed decreased heroin consumption after lentivirus injection (LV-control, n = 6, LV-miR-218, n = 9, Two-way RM ANOVA. *P < 0.05 *vs.* LV-control on the same day). Data are shown as mean ± s.e.m.

To explore the role of miR-218 in heroin-induced behavioral plasticity, we delivered recombinant lentivirus that express miR-218 (LV-miR-218) or scramble control sequence (LV-control) into the NAc region (Fig. 3a). The expression of virus-derived reporter GFP and its co-localization with neuronal marker NeuN were observed (Fig. 3b). Quantitative PCR analysis revealed that significant increase of miR-218 level could be detected 3 days after lentivirus injection and a 2-fold increase was observed 7 days after LV-miR-218 injection in NAc (Fig. 3c). To determine if miR-218 played a role in heroin-induced reinforcement, the effect of miR-218 overexpression in NAc was examined on CPP. One week after NAc infusion of LV-miR-218 or LV-control, rats were given repeated heroin injections paired with a contextually-distinct chamber in CPP apparatus, and their performance in the subsequent CPP test was scored. We found a significant effect of treatment ×test phase interaction (F_(1,17)_ = 11.80, *p* = 0.003). Moreover, Bonferroni’s *post hoc* analysis revealed that rats with miR-218 overexpression in the NAc exhibited reduced preference to the heroin-paired chamber, compared with LV-control injected rats (Fig. 4a).

Next, we carried out heroin self-administration tests to further validate the role of miR-218 in heroin seeking behavior. Rats were trained to self-administer heroin for 8 consecutive days, then subjected to LV-miR-218 or LV-control injection. Consistent with CPP results, miR-218 overexpression significantly reduced heroin acquisition compared with the control group (Fig. 4b).

### MiR-218 regulates drug addiction network and directly targets MeCP2

**Figure 5:**
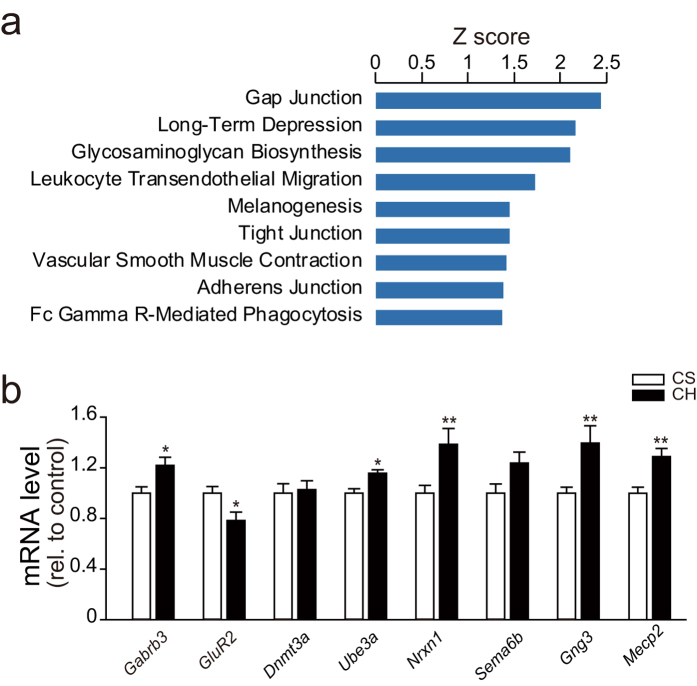
MiR-218 regulates synaptic plasticity-related genes. (**a**) MirSystem analysis of miR-218 target genes revealed significant enrichment in neuronal plasticity-related pathways. (**c**) Relative expression level of *Gabrb3, GluR2, Dnmt3a, Ube3a, Nrxn1, Sema6b, Gng3* and *Mecp2* in NAc of rats treated with saline or chronic heroin (1 mg/kg, i.p., b.i.d, CS, Chronic Saline, n = 10; CH, Chronic Heroin, n = 6; Student’s *t*-test *P < 0.05, **P < 0.01). Data are shown as mean ± s.e.m.

**Figure 6:**
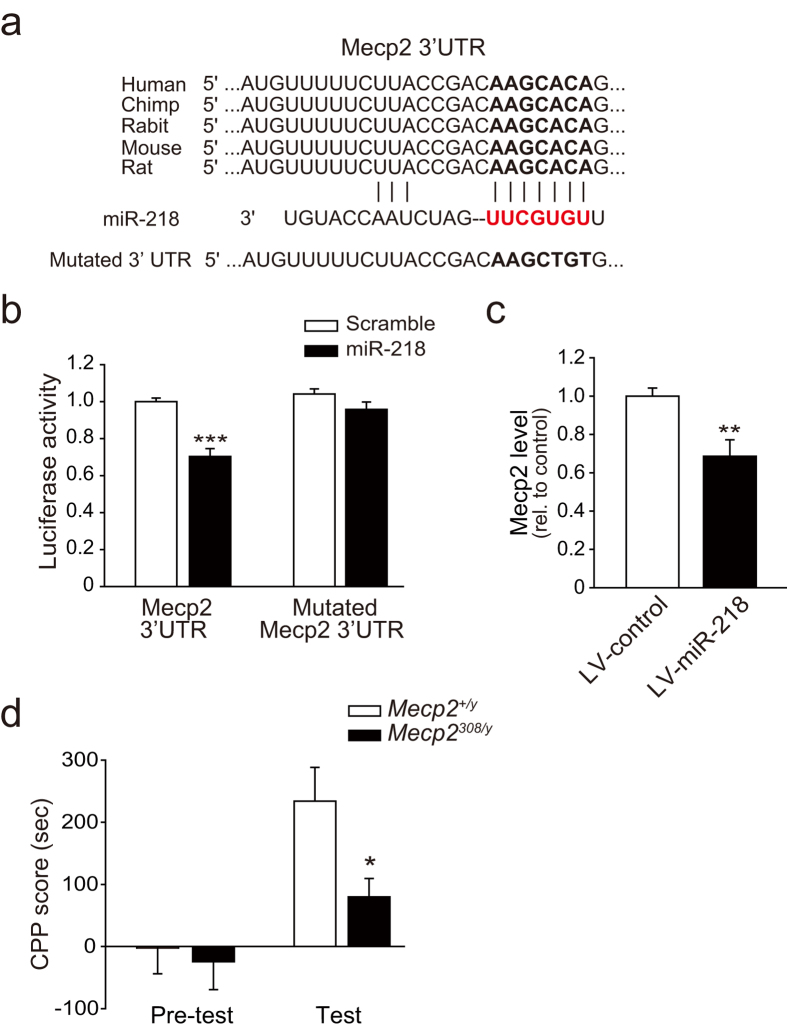
MiR-218 inhibits heroin-induced CPP by directly targeting MeCP2. (**a**) Schematic representation of miR-218 sequence and its target sequences within the 3′ UTR of *Mecp2*. The seed sequence is indicated by bold letters, while the pairing sequence of miR-218 is in red bold letters. (**b**) Relative luciferase activity with the *Mecp2* 3′ UTR or mutated 3′ UTR was determined in 293 T cells co-transfected with scramble or miR-218 mimic. (n = 6 for each group, ***P < 0.001 *vs.* scramble with *Mecp2* 3′ UTR). (**c**) Relative *Mecp2* level with LV-control or LV-miR-218 infection in C6 cell (n = 6 for each group, **P < 0.01 vs LV-control). (**d**) CPP score of *Mecp2*^+/y^ and *Mecp2*^*308/y*^ mice in pre-test and test sessions (*Mecp2*^*+/y*^, n = 9, *Mecp2*^*308/y*^, n = 7, *P < 0.05 *vs. Mecp2*^*+/y*^ mice during test session). Data are shown as mean ± s.e.m.

To infer biological roles and network communities of miR-218, miRSystem database^23^ was used for characterizing enriched functions and pathways of miR-218 targets. MiR-218 targets, co-predicted by at least two canonical databases (PicTar, TargetScan, DIANA, miRanda), were enriched in KEGG pathways highly relevant to neuronal functions, such as gap junction and long-term depression (Fig. 5a), suggesting its potential involvement in regulation of neuronal plasticity. We selected a few predicted targets of miR-218, which are indicated by both KARG database and recent literature to contribute to drug-induced neuroplasticity, and tested the validity of the miRNA-gene regulatory relationship. Indeed, Gabrb3, and Mecp2, which play critical roles in drug induced synaptic plasticity^22^,^24^,^25^,^26^, Nrxn1, Gng3, and Ube3a, which are involved in axon guidance and dendritic morphogenesis^27^,^28^,^29^, were increased in NAc after chronic heroin administration (Fig. 5b). Using luciferase reporter system, we first cloned each predicted target site into the 3′ UTR of *Renilla* luciferase and co-transfected the constructs with miR-218 mimic or scramble control in HEK293T cells. Reduced luciferase activity indicated that these genes were the directly regulated by miR-218 (Fig. S1). Among the targets identified, we focused on MeCP2 for its crucial role in psychostimulus-induced plasticity^22^ and broad transcription regulatory activity^30^. As shown in Fig. 6a, the predicted miR-218 target sequences of *Mecp2* were highly conserved among mammals. Target sequence of *Mecp2* 3′ UTR and a seed region-mutated sequence were subjected to luciferase assay. We found that the expression of luciferase with mutant *Mecp2* 3′ UTR was not affected by miR-218 mimic transfection, in contrast to significantly attenuated activity of luciferase with wild type *Mecp2* 3′ UTR (Fig. 6b). Furthermore, overexpression of miR-218 in C6 cells downregulated *Mecp2* expression level (Fig. 6c), which further confirmed that *Mecp2* is a target of miR-218. Coincident with miR-218 overexpression result, heroin CPP was significantly blocked in *Mecp2*^*308/y*^ mice (Fig. 6d). These data support the notion that miR-218 directly targets *Mecp2* in a sequence-specific manner and thus inhibits heroin seeking behavior.

## Discussion

MiRNA functions are executed by the multiprotein RNA-induced silencing complex containing Argonaute (Ago)^31^. The expression level of Ago2 is decreased in the VTA of morphine-dependent rats^32^. Ablation of Ago2 in dopamine 2 receptor-expressing neurons attenuates the reinforcing effects of cocaine and cocaine induced CPP, implicating miRNA may contribute to addictive behaviors^16^. Hollander *et al*. reported cocaine SA increase miR-212 in striatum and increasing miR-212 in this region decrease cocaine reward^18^. Chronic cocaine exposure also regulates miR-124 and miR-181a expression in brain, and overexpression of miR-124 in NAc reduced cocaine CPP while miR-181a has the opposite effect^33^. He *et al*. reported let-7 targets the 3′ UTR of MOR, and chronic morphine treatment increased let-7 expression in brain, while let-7 inhibition causes increased levels of MOR and partially attenuated opioid antinociceptive tolerance^34^. MiR-382 modulated the expression of ΔFosB which had been linked directly to several addiction-related behaviors, and overexpression of miR-382 in NAc decreased voluntary intake and preference for alcohol in rats^35^. Our research found that miR-218 in NAc decreased by heroin administration and overexpression of miR-218 inhibited heroin seeking behavior, providing a direct evidence of miR-218 involved in heroin induced behavioral plasticity.

In the present study, we found that miR-218 was downregulated by chronic heroin use in NAc. Consistently, bioinformatic analysis indicated the predicted targets of miR-218 was enriched in addiction-related genes and involved in neuroplasticity. MiR-218 is encoded by an intron of the Slit gene and inhibits the expression of Robo1 which play important roles in axonal growth^36^,^37^. Recent studies indicated that miR-218 plays a crucial role in motor neuron differentiation and loss of miR-218 cause systemic neuromuscular failure^38^,^39^. Recent large-scale miRNA expression profiling showed that miR-218 exhibits ubiquitous enrichment in hippocampus, cortex and cerebellum, but exhibits lower expression in olfactory bulb and brain stem^40^. Hippocampal cell-type-based analysis of miRNAs revealed that miR-218 is specifically enriched in neurons, compared with microglia, astrocytes and oligodendrocytes^41^. And more specifically, it is enriched in parvalbumin (PV)-positive, but not neuropeptide somatostatin expressing GABAergic interneurons. And its expression in CaMKIIα positive neurons exhibits three-fold higher expression than Gad2-expressing neurons^42^. Together with the predicted pathway of its target genes, we postulate that miR-218 is essential to the regulation of neuronal plasticity.

MeCP2 is a transcription factor binding to methylated cytosine residues in DNA and recruits histone deacetylases and other transcriptional repressors to silence target genes^43^. Previous studies reported a critical role of Mecp2 in normal neurological function. Hippocampal glutamatergic neurons that lack MeCP2 display a reduction in synaptic response whereas neurons with doubling of Mecp2 exhibit a two-fold enhancement in synapse number^44^. Mutations in MeCP2 results in nonsyndromic mental retardation, mild learning disability, and classic autism^45^,^46^,^47^. Recent studies reveal the role of MeCP2 in psychostimulant induced behavioral plasticity. Extended cocaine access increased Mecp2 expression in dorsal striatum and striatal Mecp2 knockdown decrease cocaine intake^22^. Coincidently, we found reduced heroin conditioned preference in *Mecp2*^*308/y*^ mice. However, Deng *et al*. found that *Mecp2*^308/y^ mutant mice showed enhanced locomotor response to acute amphetamine administration and impaired CPP, but lentivirus mediated knockdown or overexpression of MeCP2 in NAc enhanced or inhibited amphetamine-induced locomotion and CPP respectively^48^. Later, the same group found *Mecp2* Ser421Ala knock-in mice display both a reduced threshold for amphetamine sensitization and enhanced behavioral sensitivity to the reinforcing properties in cocaine self-administration, indicating that MeCP2 in NAc limited behavioral plasticity upon repeated psychostimulant exposure^49^. Functionally, early studies found that MeCP2 interacts specifically with methylated cytosine in the CG context (mCG). However, recent evidence suggest that MeCP2 regulates expression of genes in a spatial- and temporal- distinct manner and binds to non-CG methylation^30^,^50^,^51^. These result implicated that the role of MeCP2 played in drug addiction seems much more complicated, and more detail understanding the molecular mechanism of MeCP2 in drug induced plasticity still need to be experimental verified.

Taken together, our results reveal that miR-218 is involved in heroin induced behavioral plasticity possibly by regulating Mecp2. Overexpression of miR-218 in NAc decreased heroin conditioned preference and heroin self-administration. MiR-218 is likely to contribute to heroin related modifications, both transcriptionally and epigenetically. Our results provide a promising and novel approach to the treatment of heroin addiction. However, the role of miR-218 in other drug induced plasticity and the downstream signaling pathways regulated by miR-218 in addiction are remained to be elucidated.

## Methods

### Subjects

Male Sprague-Dawley rats were purchased from the Shanghai SLAC Laboratory Animal Co. Ltd. and housed with a 12 h reverse dark/light cycle at 23 °C. All subjects were allowed free access to food and water. Rats used for experiments were 8–10 weeks old. *Mecp2*^*308/y*^ mice and littermates were maintained by mating heterozygotes with C57BL/6 J. All animal treatments were in strict accordance with the National Institutes of Health Guide for the Care and Use of Laboratory Animals and were approved by Animal Care and Use Committee of Shanghai Medical College of Fudan University.

### Lentivirus Construction and Packaging

The lentivirus construct and packaging were performed as we described previously^6^. Briefly, human U6 promoter and small hairpin RNA that overexpress miR-218 (LV-miR-218, TTGTGCTTGATCTAACCATGT) or control sequences (LV-control, GCAGTTATCACGTCTATGTTT) were cloned into FG12 plasmid and co-transfected into HEK293T cells with pMDLg/RRE, pRSV/rev and pHCMV-G for lentivirus packaging. Forty-eight hours after transfection, the culture medium was collected and concentrated by ultracentrifugation, titrated, aliquoted, and stored at −80 °C, the final virus titer was estimated to be 1 × 10^8^ IU/ml.

### Intravenous heroin self-administration

To facilitate subsequent heroin self-administration, rats were trained to press an active lever for food pellets until they self-administered 100 pellets for 3 consecutive days on fixed-ratio 1 program. Then the rats were anesthetized, and a silastic catheter (0.04 cm interior diameter) was inserted 3 cm into the jugular vein, and the other end of the catheter was attached to a stainless steel pedestal and mounted on the skull. Bilateral 26-gauge guide cannulae were implanted in NAc (1.7 mm A/P, ±1.6 mm M/L, and −5 mm D/V). After surgery, the rats were allowed to recover for 7 days and the catheters were flushed daily with saline supplemented with heparin (30 IU/ml) and gentamycin (5 mg/ml). Next the rats were allowed to self-administer heroin (0.05 mg/kg/injection, FR1 program) during a daily 4 h session. After the 8th session, the rats were anesthetized and subjected to lentivirus infusion with needle (Plastics One, Inc.) that extend 1.5 mm beyond the guide cannulae. The self-administration procedure was resumed 3 days after lentivirus injection and tested on FR1 schedule for six consecutive days.

### Conditioned place preference

The apparatus was three-chambered (Med Associates), with two large side chambers with distinct floors and decorations and one small center chamber. In pretest, the rat was first confined in the center chamber for adaption for 5 min, and then allowed to freely explore the chambers for 15 min. Unbiased rats were subjected to lentiviral injection into NAc (1.7 mm A/P, ± 1.6 mm M/L, and −6.5 mm D/V). The rats were allowed to recover for 7 days. Then the rats were alternatively confined in each large chamber for 30 min after injection of heroin (1 mg/kg, i.p., days 1, 3, 5) or saline (days 2, 4, 6). On the test session (day 8), the rats were adapted in center chamber for 5 min and allowed to explore all chambers unrestrictedly for 15 min. The time which the rat spent in each large chamber was recorded and CPP score was defined as the time spent in the heroin-paired chamber minus the time spent in the saline-paired chamber during the CPP test. The CPP pretest, conditioning and test procedure of *Mecp2*^*308/y*^ and *Mecp2*^*+/y*^ mice followed the same procedure.

### MiRNA microarray and data analysis

Total RNA are harvested using TRIzol (Invitrogen) and RNeasy mini kit (QIAGEN) according to manufacturer’s instructions. The RNA samples are labeled using the miRCURY™ Hy3™/Hy5™ Power labeling kit and hybridized on the miRCURY™ LNA Array (v.11.0). Scanning was performed with the Axon GenePix 4000B microarray scanner. GenePix pro V6.0 is used to read the raw intensity of the image. Median normalized signal of each miRNA was used to represent expression level. MiRNAs that exhibit 1.25-fold up-regulation or 0.75-fold down-regulation was chosen for further analysis.

Network propagation based model was generated as described^19^. Briefly, miRNA families that are highly conserved (conservation score ≥2) were chosen from TargetScan Human 7.1, and their predicted targets were used. Curated human transcriptional regulation interactions database (HTRIdb) was provided by the software. Genes from the KARG database was used, and their corresponding Reliability score was log transformed and used as candidate list for correlation (i.e. NPES) calculation with the algorism. Leading edge genes of each miRNA was extracted, with NPES score at or before the point of maximum NPES score from the ranked list. Pathway analysis was performed with miRSystem database with default settings. Microarray data have been deposited into the Gene Expression Ominibus with accession number GSE89202.

### Quantitative real-time PCR

TaqMan small RNA assay (Applied Biosystems) for miRNAs of interest were performed. U6 was used for normalization. Primers used in RT-PCR were Mecp2 [CCTATGTATGATGACCCCACCT (forward), GAAAGGCATCTTGACGAGAAGT (reverse)], Gabrb3 [ATGGAACAGTGCTGTACGGG (forward), ACCTGTGGCGAAGACAACAT (reverse)], GluR2 [CCTTTATGCGGCAAGGATGC (forward), AGGGCTCTGCACTCCTCATA (reverse)], Dnmt3a [TGGTGTGTGTCGAGAAGCTC (forward), TTCGTAGATGGCTTTGCGGT (reverse)], Ube3a [ACCCTGATGTCACCGAATGG (forward), TCATTCGTGCAGGCCTCATT (reverse)], Nrxn1 [GCATCATCACAGAACGACGC (forward), GGATCCGCGATGATGTTCCT (reverse)], Sema6b [ATGGGATGCTCTTCACAGCC (forward), ATTCTTGCATACACGGGCCA (reverse)], Gng3 [CAACGCCTGTGTTAGCGTTC (forward), GCTTTCATTGCACGCTCGTT (reverse)], β-actin [CAACCTTCTTGCAGCTCCTCCGT (forward), AGGGTCAGGATGCCTCTCTTGCTC (reverse)]. All samples were run in duplicate. The relative expression level of mRNA was calculated by 2^−ΔΔCt^ method.

### Luciferase assay

Predicted target sequences of miR-218 were cloned to the 3′ UTR of *Renilla* luciferase of psiCHECK-2 vector. The target sequences were, Gabrb3 [CCTTTATTTCTGTACTAACTTATCTCATAAGCACACCCAATTCCTCCTAG], GluR2 [TATTGTTAGTCTCTTGATTCATAATGACTTAAGCACACTTGACATCAACT], Dnmt3a [TTGGTTGTCTCTAGCCTGATCAGATAGGAGCACAAACAGGAACAGAATAG], Ube3a [TCTTTGTAGCTGGACAGCACAATGTTTATGATTTATTTAATCTGTAGTTT], Nrxn1 [AAACTTATTTACTTTCCTTTTTATGAAGCACATACAAAAGAAGACAGGGA], Sema6b [CGGGTGGGGATCTCCTCGCCACAGGGAAGCACAAGAGCCCCCTCCATCCC], Gng3 [CGCACTTATCCTGAGATTATCTGAAGCACAAGGCCCTCCTTACCCACCTC]. Mecp2 [TTGGGATGTTTTTCTTACCGACAAGCACAGTCAGGTTGAAGACCTAACCA]. We transfected 500 ng recombinant vector with 10 pmol miRNA mimic or scramble (miR-218, TTGTGCTTGATCTAACCATGT, Scramble, GCAGTTATCACGTCTATGTTT) into HEK293T cells with Lipofectamine 2000 (Invitrogen). After 24 h, dual-luciferase report assay system (Promega) was performed to measure *Renilla* and *Firefly* luciferase activity, then *Renilla* activity was normalized to the *Firefly* luciferase activity. All the experiments were repeated for at least three times.

### Immunofluorescence staining

The rats were anesthetized and transcardially perfused with saline, followed by 4% paraformaldehyde in PBS. Then the brains were quickly removed and post fixed with 4% paraformaldehyde at 4 °C for about 4 h and stored in 30% PBS-buffered sucrose solution for 72 h. The brains were sectioned (40 μm) with a cryostat (Leica) and the sections were washed in PBS, blocked with blocking buffer (10% donkey serum in PBS containing 0.3% Triton X-100) for 1 h, then incubated with NeuN antibody (1:500, rabbit polyclonal, Upstate Biotechnology) at 4 °C overnight. Sections were subsequently rinsed with PBS and incubated for 1 h at room temperature with CY3 goat anti-rabbit antibody (1:1000, Vector Laboratories). The images were captured with a microscopic CDD camera (Olympus).

### Statistical analysis

Data are presented as mean ± s.e.m. For quantitative PCR results, data were analyzed by student’s *t*-test or one-way analysis of variance (ANOVA). For all the behavior results, data were analyzed by two-way repeated measures ANOVA followed by Bonferroni’s *post hoc* test. Luciferase activity data were analyzed by student’s *t*-test or two-way ANOVA followed by Bonferroni’s *post hoc* test. *P* < 0.05 are defined as statistically significant.

## Additional Information

**How to cite this article:** Yan, B. *et al*. MiR-218 targets MeCP2 and inhibits heroin seeking behavior. *Sci. Rep.* **7**, 40413; doi: 10.1038/srep40413 (2017).

**Publisher's note:** Springer Nature remains neutral with regard to jurisdictional claims in published maps and institutional affiliations.

## Supplementary Material

Supplementary Figures and Tables
