# Supplementary material for: MiR-218 targets MeCP2 and inhibits heroin seeking behavior
Source: Sci Rep. 2017 Jan 11;7:40413. doi: 10.1038/srep40413 (PMC5225456; doi:10.1038/srep40413)
Supplement: Supplementary Figures and Tables [file srep40413-s1.pdf]

MiR-218 targets MeCP2 and inhibits heroin seeking behavior

Biao Yan[1,2], Zhaoyang Hu[1,2], Wenqing Yao[1,2], Qiumin Le[1,*], Bo Xu[1], Xing Liu[1], and Lan Ma[1,*]

[1]The State Key Laboratory of Medical Neurobiology, Institutes of Brain Science and The Collaborative Innovation Center for Brain Science, Fudan University, 138 Yixueyuan Road, Shanghai 200032, China.

[2]Theses authors contributed equally to this work.

*Correspondence to: lanma@fudan.edu.cn or qle09@fudan.edu.cn

Fig. S1

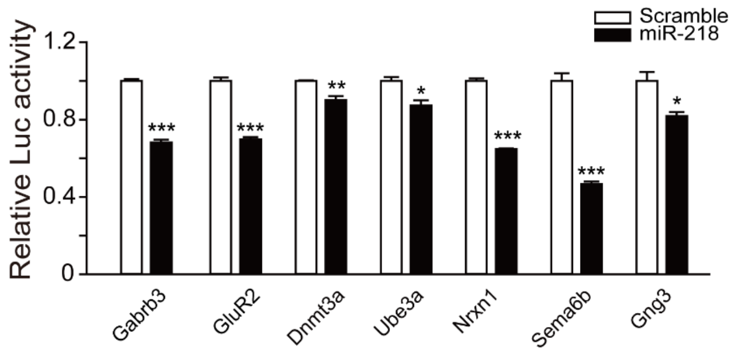

Supplementary Figure 1: Luciferase validation of potential miR-218 targets in addiction-related pathway. Relative luciferase activity with the Gabrb3, GluR2, Dnmt3a, Ube3a, Nrxn1, Sema6b, Gng3 3´ UTR in 293T cells co-transfected with scramble or miR-218 mimic synthetic RNA (n = 3 for each group, *P < 0.05, **P < 0.01, ***P < 0.001 vs scramble).

Supplementary Table 1. Putative addiction-related miRNA families identified by NP method.

Symbol	Z score	Targets	maxNPES	Leading edge targets
**miR-128-3p**	1.5027	764	0.312	*Mapk1, Stat1, E2F3, Grm5, Gria1, Gabbr2, Sp1, Cacna1A, Cnr1, Gad1, Creb1, Fosb, Pparg, Ppp1Cc, Slc6A1, Grin2D, E2F6, Nfat5, Lrrfip1, Nptx1, Kcna1, Syt1, Ncam1, Nova1, Rap1B, Gabrb1, Rps6Ka5, Grb2, Nf1, Mapk14, Atp2B1, Thrb*
**miR-181-5p**	0.0993	758	0.3046	*Mapk1, Fos, Gria2, Yy1, Grm5, Sp1, Drd1, Gnaq, Cnr1, Creb1, Map2K1, Bcl2, Homer1, Nfat5, Lrrfip1, Tnf, Ppp1Cb, Nr3C1, Mef2A, Ywhag, Hspa5, Atxn3, Cntn4, Nova1, Rap1B, Gabrb1, Mapt, Gnao1, Grid1, Atp2B1, Gabra1, Gabra4, Dusp6, Thrb, Dusp5, Zeb1*
**miR-9-5p**	0.2388	959	0.2716	*Drd2, Irf1, Abat, Srf, Gabbr2, Gabrb2, Gad1, Prkca, Cnp, Nptx1, Scn2B, Kcna1, Gch1, Atxn3, Syt1, Cntn4, Rap1B, Nfkb1, Atf1*
**miR-27-3p**	0.4497	860	0.2603	*Stat1, Grm5, Camk2A, Slc1A2, Sp1, Cacna1A, Creb1, Fosb, Pparg, Ppp1Cc, Slc6A1, Grin2D, Camk4, E2F6, Nfat5, Syt1, Ncam1, Nova1, Gabrb1, Rps6Ka5, Mapt, Grb2, Nf1, Mapk14, Atp2B1, Thrb, Dusp5, Tbp*
**miR-129-5p**	0.6052	326	0.2459	*Mapk1, Stat1, Grm5, Gabbr2, Cacna1A, Gnaq, Glul, Kdm2B, Camk4, Mef2A, Nptx1, Syt1, Rps6Ka5, Ybx1, Sp3*
**miR-218-5p**	0.7449	677	0.2393	*Gria2, Yy1, Tac1, Sp1, Gabrb2, Creb1, Ppp1Cc, Slc6A1, Lrrfip1, Ppp1Cb, Kcnd2, Scn2B, Kcna1, Syt1, Gnai2, Plcg1, Gnao1, Thrb, Dusp5*
**miR-135-5p**	0.0898	528	0.2281	*Sp1, Gabrb2, Slc6A4, Plcb1, Ppp1Cc, Nfat5, Lrrfip1, Elk3, Adcyap1, Mef2A, Nptx1, Ywhag, Scn2B, Syt1, Gabrb1, Plcg1, Rps6Ka5, Mapk10, Sp3, Nefm, Cacna1D, Thrb, Dusp5, Dusp8, Mef2C, Tfap2A*
**miR-148-3p/152-3p**	0.8809	450	0.2244	*E2F3, Grin2A, Camk2A, Cnr1, Fosb, Kdm2B, Homer1, Nfat5, Ppp1Cb, Nptx1, Atxn3, Syt1, Ncam1, Cntn4, Nova1, Rps6Ka5, Gadd45A, Erbb3, Dusp8*
**miR-34-5p/449-5p**	0.7546	425	0.217	*Mycn, E2F3, Yy1, Htr2C, Gnaq, Map2K1, Bcl2, Crhr1, Slc6A1, Prkacb, Camk4, Scn2B, Syt1, Gnai2, Casp2, Gabrb1, Plcg1, Mapt, Gnao1, Grid1*

**miR-144-3p**	0.5218	588	0.2166	*Mycn, Fos, Grm5, Gabrb2, Creb1, Fosb, Tal1, Egfr, Nr3C1, Mef2A, Nptx1, Adrb1, Kcna1, Nova1, Rap1B*
**miR-23-3p**	0.0427	702	0.2117	*Irf1, Stat1, Grm5, Htr2A, Htr2C, Fosb, Crebbp, Pgr, Ppp3Ca, Lpar1, Ppp1Cb, Adcyap1, Mef2A, Ywhag, Atxn3, Rap1B, Ptk2B, Dusp5, Zeb1, Pten, Mef2C, Stat5B*
**miR-153-3p**	2.0622	563	0.2115	*E2F3, Htr1F, Plcb1, Bcl2, Crebbp, Kdm2B, Syn1, Ppp3Ca, Mef2A, Kcna1, Syt1, Nova1, Igf1R, Rps6Ka5, Ryr3, Sp3, Grb2, Nf1, Snca, Pten, Mef2C, Fyn, Myb*
**miR-204-5p/211-5p**	1.1274	338	0.2052	*Mapk1, Gabbr2, Gnaq, Creb1, Bcl2, Nfat5, Nr3C1*
**miR-145-5p**	0.1917	472	0.1894	*Mycn, E2F3, Slc1A2, Htr2A, Sp1, Dlg4, Pmp22, Ppp3Ca, Adcyap1, Plce1, Ywhag, Cntn4, Igf1R, Sp3, Cacna1D, Dusp6, Gdnf*
**miR-139-5p**	0.2443	244	0.1872	*Fos, Grin2A, Gabbr2, Jun, Homer1, Pmp22, Nfat5, Nptx1, Ywhag, Ppp2Ca, Rap1B, Igf1R,*
**miR-142-5p**	0.1518	539	0.1821	*Mycn, Fos, Yy1, Srf, Camk2A, Htr2A, Drd1, Nfat5, Ppp3Ca, Arhgap35*
**miR-22-3p**	0.7664	319	0.1813	*Grm5, Gabbr2, Sp1, Creb1, Slc6A1, Homer1, Nr3C1, Faslg, Mapk14, Erbb3*
**miR-24-3p**	0.6480	345	0.1807	*Gabbr2, Sp1, Gad1, Hap1, Prkca, Nfat5, Nptx1, Rap1B, Nefm, Mapk14, Erbb3, Il1R1, Dusp8, Per1*
**miR-143-3p**	2.0258	225	0.1707	*Htr2A, Htr2C, Cacna1A, Bcl2, Nova1, Igf1R, Erbb3*
**miR-7-5p**	0.8035	308	0.1673	*Grin2A, Sp1, Plcb1, Raf1, Gabbr1, Scn2B, Vdac1, Snca, Gabra1, Vdac3*
**miR-132-3p/212-3p**	0.7259	255	0.1631	*Mapk1, Slc6A1, Nfat5, Adcyap1, Mef2A, Ywhag, Nova1, Mapt, Wt1*
**miR-138-5p**	0.1298	393	0.1604	*Creb1, Rela, Casp3, Ywhah, Arhgap35, Nptx1, Per2, Scn1B, Gnai2, Nova1, Gabrb1, Grid1, Thrb, Mapk6*
**miR-10-5p**	0.5119	203	0.1486	*E2f3, Bdnf, Gabrb2, Creb1, Nfat5*
**miR-338-3p**	0.8484	221	0.1452	*Stat1, Camk2A, Gnaq, Kcnd2, Nova1, Mapt, Ppm1B*
**miR-193-3p**	9.1819	133	0.1442	*Mycn*
**miR-155-5p**	0.9142	252	0.1442	*Fos, Bdnf, Rela, Nfat5, Mef2A*
**miR-221-3p/222-3p**	0.9929	260	0.1396	*Fos, Nfat5*
**miR-194-5p**	0.4426	244	0.1363	*Stat1, E2F3*
**miR-365-3p**	1.3728	178	0.1245	*Bdnf, Ywhah, Nr3C1, Cntn4*
**miR-140-5p**	0.0067	212	0.1228	*Bcl2L1, Ppp1Cc, Nfat5, Ywhag, Rap1B, Igf1R*

**miR-31-5p**	0.6497	210	0.1144	*Slc1A2, Homer1, Ppp3Ca*
**miR-210-3p**	0.8070	19	0.1047	*Bdnf, Mef2A*
**miR-150-5p**	0.0408	142	0.1003	*Sp1, Prkca, Ppp2Cb, Nefm, Gabrg2, Zeb1, Myb*
**miR-146-5p**	0.5895	120	0.0984	*Kdm2B, Syt1, Nova1, Grid1, Gdnf, Per1, Rel*
**miR-208-3p**	0.6935	133	0.0942	*Ppp3Cb, Scn2B, Nova1, Ybx1, Sp3, Gabra1, Mapk6*
**miR-18-5p**	0.0425	166	0.0941	*Prkacb, Nfat5, Nr3C1, Kcna1, Igf1, Rasa2,*
**miR-223-3p**	0.9148	178	0.0918	*Mpz, E2F1, Rbpj, Igf1R, Sp3, Atp2B1, Zeb1, Mef2C*
**miR-490-3p**	0.4216	96	0.0509	*Slc1A3*
**miR-99-5p/100-5p**	-3.8616	38	0.048	*Ppp1Cb, Igf1R*
